# A case of bloodstream infection caused by *Neisseria gonorrhoeae*

**DOI:** 10.1515/biol-2022-1017

**Published:** 2025-04-10

**Authors:** Danhui Kong, Yue Qiu, Limin Zou, Qi Zhang, Wenjun Zhou, Jie Yang

**Affiliations:** Department of Orthopedics, The People’s Hospital of Danyang, Danyang Hospital Affiliated to Nantong University, Danyang, Jiangsu, 212300, China; Clinical Laboratory, The People’s Hospital of Danyang, Danyang Hospital Affiliated to Nantong University, Danyang, Jiangsu, 212300, China

**Keywords:** *Neisseria gonorrhoeae*, bloodstream infection, fastidious bacteria, VITEK mass spectrometry

## Abstract

*Neisseria gonorrhoeae* is fastidious, visual etiological evidence is extremely difficult to obtain, and positive results from blood cultures are even rarer. A 51-year-old female patient was admitted to our hospital for 3 days with fever, and polyarticular pain was diagnosed as infectious fever by a series of examinations, but the pathogen could not be determined. *N. gonorrhoeae* was identified by blood culture and mass spectrometry. Combined with the drug sensitivity test results, ceftriaxone was given and she recovered. This case is extremely rare, underlining the importance of standardized timely testing and collaboration between clinical and microbiology laboratories.

## 1 Introduction

Bloodstream infection is a life-threatening systemic infectious disease with diverse pathogenic microorganisms. At present, positive blood culture is still the gold standard for the diagnosis of bloodstream infection. *Neisseria gonorrhoeae* (gonococcus) and *Neisseria meningitidis* (meningococcus) are important global pathogens that cause the sexually transmitted diseases gonorrhea and meningitis, respectively, as well as sepsis [1]. *N. gonorrhoeae* is transmitted primarily through sexual activity; it mainly invades the columnar epithelium of the human urogenital tract, destroys the mucosa, and invades the submucosa, causing acute or chronic suppurative inflammation [2,3]. A 26-year-old woman was found to have disseminated gonococcemia [4]. However, cases of bloodstream infection caused by *N. gonorrhoeae* are rare. To support this case study, a case of bloodstream infection due to *N. gonorrhoeae* is reported, hoping to provide some references for clinical diagnosis and treatment.

## 2 Clinical data

A 51-year-old female patient was admitted due to “fever with polyarticular pain for 3 days” on December 20, 2023. Present medical history: The patient experienced chills and fever without obvious inducement 3 days ago, accompanied by headache, which was improved after self-administration of ibuprofen, and then developed pain in the back of the left foot and the left wrist, swelling pain in the left wrist and the right wrist, pain when moving both knees and backs of the feet, obvious morning stiffness of hands, more than half an hour every day, and no improvement after rest at home.

Cervical magnetic resonance showed posterior protrusion of C3/4, C4/5, C5/6, and C6/7 intervertebral discs, thickening of C5/6 ligamentum flavum, and spinal stenosis. Admission examination: Albumin 35.0 (g/L), blood glucose 7.04 (mmol/L), urea 8.42 (mmol/L), creatinine 99.8 (μmol/L), triglyceride 2.18 (mmol/L), rheumatism three items: antistreptolysin O 80.80 (IU/mL), rheumatoid factor <20.00 (IU/mL), C-reactive protein 183.00 (mg/L), blood cell analysis: white blood cell (WBC) count 14.89 (10 × 10^9^/L), neutrophil percentage 85.10 (%), lymphocyte percentage 7.60 (%), hemoglobin amount 72 (g/L), platelet count 376 (10 × 10^9^/L), and erythrocyte sedimentation rate 88 (mm/h). Immunoglobulin and lymphocyte subsets were normal. For further diagnosis and treatment, the outpatient department was admitted to the hospital with “joint pain to be investigated.”

Previous history: diabetes mellitus and two cesarean sections. The patient’s immune status was normal. No history of pelvic inflammatory disease. Personal history: denied sexually transmitted diseases and sexual intercourse. Diagnosis on admission: infectious fever, arthralgia, cause to be investigated, moderate anemia, type 2 diabetes, cervical spondylosis.


**Informed consent:** Informed consent was obtained from all individuals included in this study.
**Ethical approval:** The research related to human use has been complied with all the relevant national regulations and institutional policies and in accordance with the tenets of the Helsinki Declaration, and has been approved by the authors’ institutional review board or equivalent committee.

## 3 Laboratory tests

**Figure 1:**
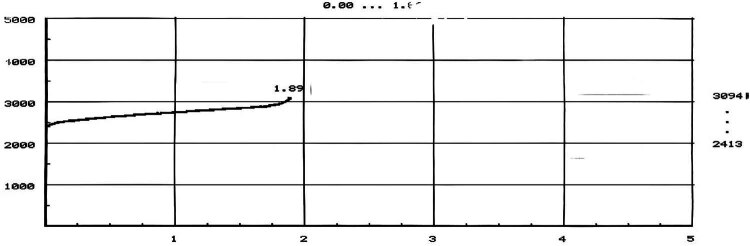
Growth curve of bacteria.

**Figure 2:**
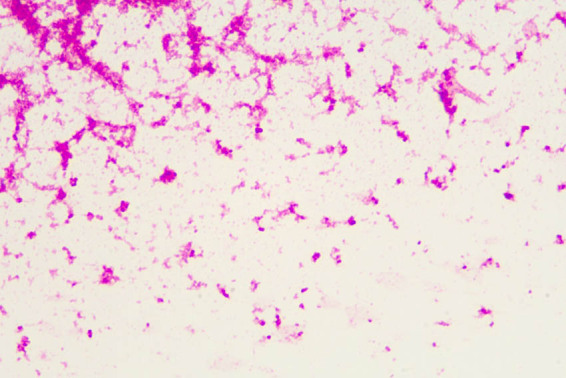
Microscopic observation and identification of bacteria after staining.

Procalcitonin 0.681 (ng/mL); high, indicating infection; ferritin, folic acid and vitamin B12 were not significantly abnormal; urinary sediment quantification and urinalysis: WBC count 356.4 (/µL), indicating urinary tract infection; blood culture was taken immediately after admission, and positive results were reported on the third day of culture by Merieux automatic blood culture instrument (BACT/ALERT 3D). Figure 1 shows the growth curve. Gram-negative renal diplococcus was detected by smear staining microscopy [5], as shown in Figure 2.

**Figure 3:**
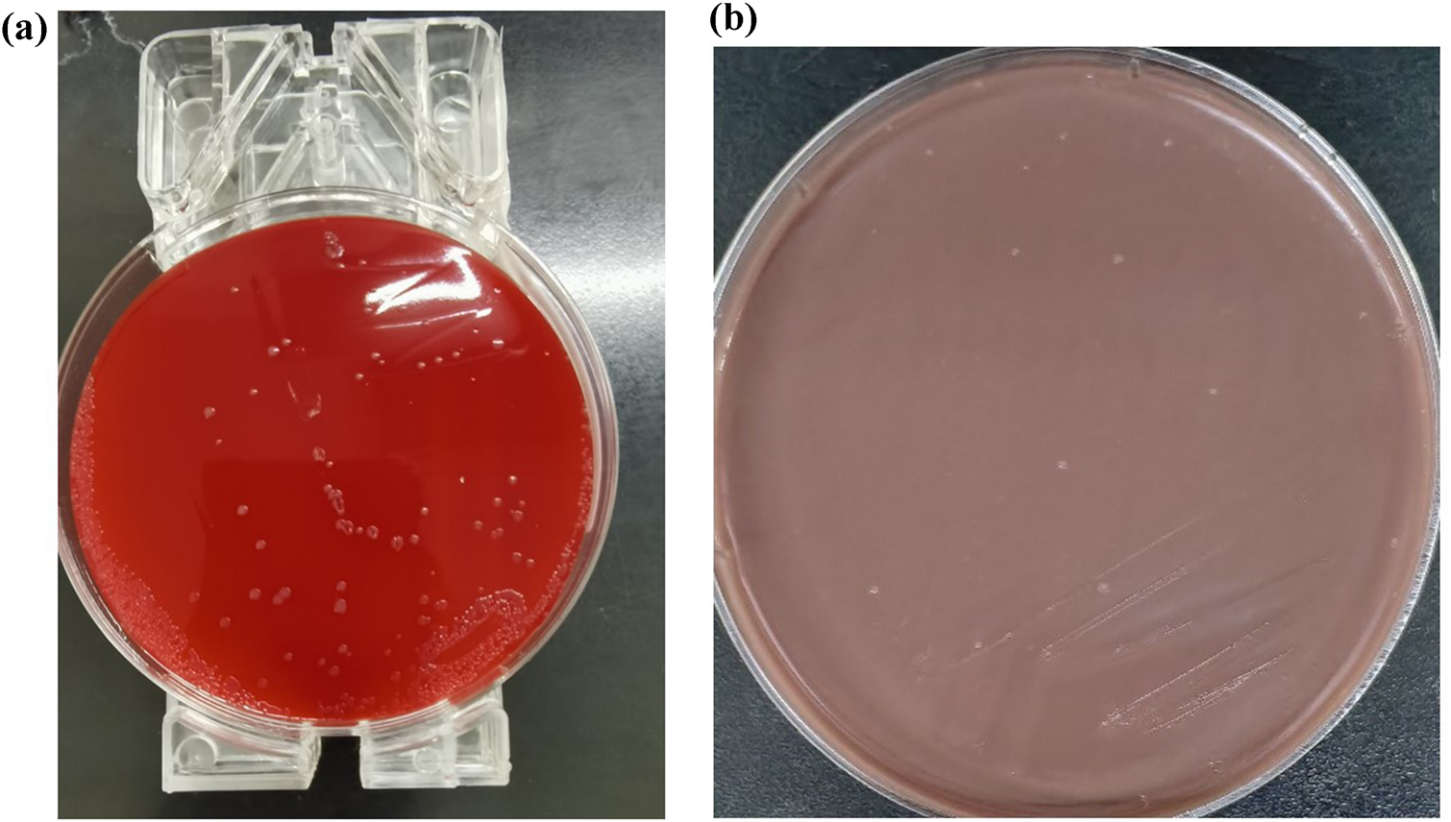
Culture results of blood agar plate (a) and chocolate agar (b).

**Figure 4:**
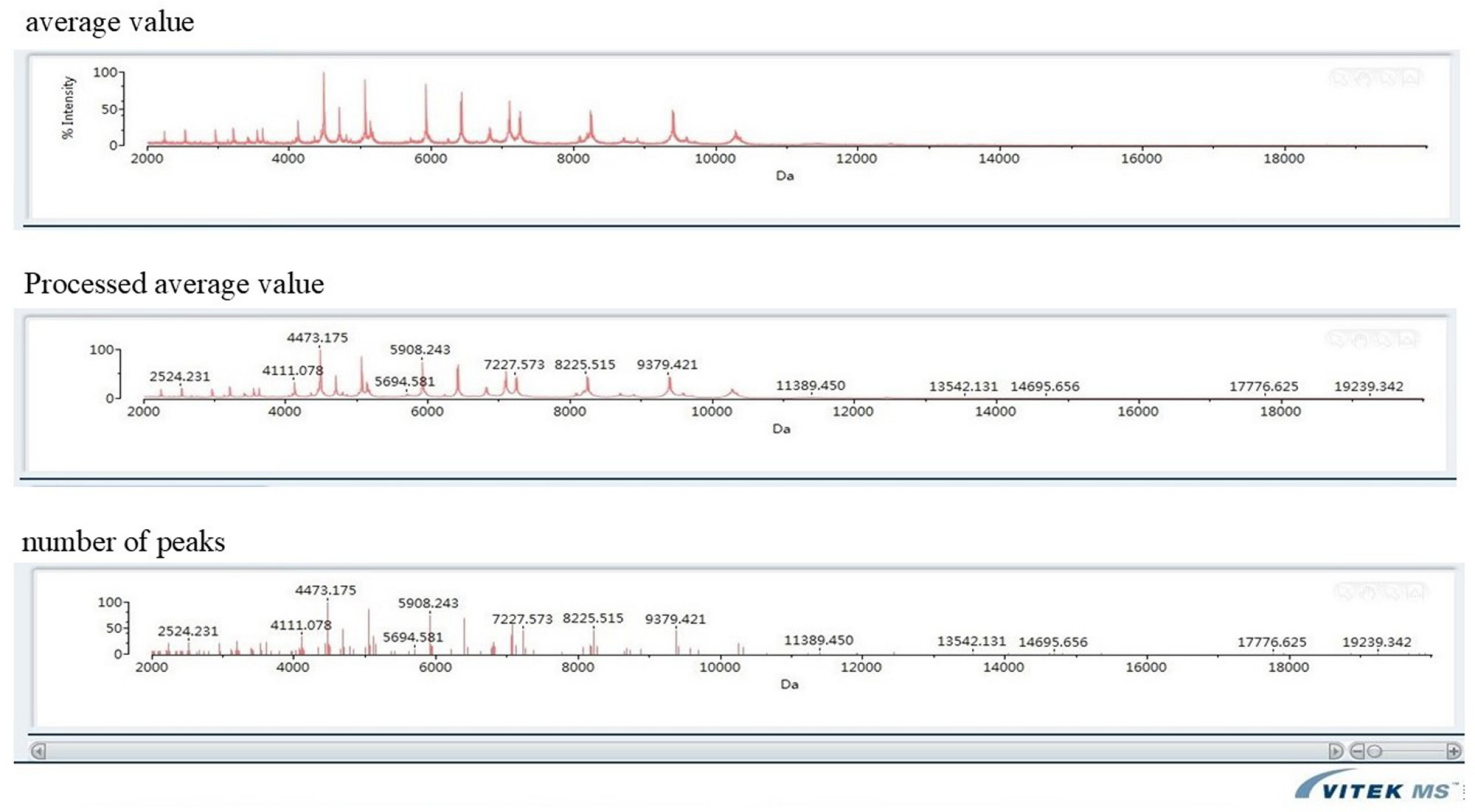
Identification of *N. gonorrhoeae* and results of mass spectrometry identification.

Urine culture and vaginal secretion culture were also recommended, but *N. gonorrhoeae* was not detected in the patient’s urine and vaginal secretions. The blood culture was transferred to blood agar plate and chocolate agar, and transparent, moist, and protruding microcolonies could be seen when cultured in 5% CO_2_ at 37°C (Figure 3). The bacteria-coated target plate was identified as *N. gonorrhoeae* by Merieux mass spectrometer matrix-assisted laser desorption ionization time-of-flight mass spectrometry (MALDI-TOF) with a confidence interval of 99.9% (Figure 4). Disk diffusion method (Kirbv-Bauer method) was used to detect the susceptibility of strains to antibacterial drugs (Table 1).

**Table 1:** Drug sensitivity of strains

Antibiotic	Breakpoint	Radial diffusion (mm)	Result
Ceftriaxone	S: ≥35	36	S
Cefotaxime	R: ≤22; S: ≥26	34	S
Cefepime	S: ≥31	34	S
Azithromycin	S: ≥30	30	S
Ciprofloxacin	R: ≤27; S: ≥41	12	R
Tetracycline	R: ≤30; S: ≥38	33	I

## 4 Discussion

*N. gonorrhoeae* is a pathogenic bacteria, and humans are its only natural host, mainly through sexual transmission, causing urinary tract, cervical, rectal, pharyngeal, and eye mucosal infections [6,7,8]. However, this case and her husband denied sexual history. At present, the diagnosis of *N. gonorrhoeae* infection methods mainly includes smear staining microscopy, culture, and molecular biology detection. *N. gonorrhoeae* was identified by smear staining, culture, and MALDI-TOF in our hospital, especially in blood culture, which was the first case. Bloodstream infection refers to disseminated infection caused by pathogenic microorganisms entering the bloodstream [9,10]. It is a systemic infectious disease endangering human life. The main pathogenic microorganisms include bacteria, fungi, and viruses, which can lead to bacteremia and sepsis. In severe cases, it can cause shock, disseminated intravascular coagulation, multiple organ failure, and even death [11,12].

At present, the gold standard for the diagnosis of bloodstream infection shows still positive blood culture. For patients suspected of infection, it is very important to keep blood culture at the first time [13,14]. In this case, blood culture was taken before antibiotics were used. When the instrument alarm was positive, the smear staining result was gram-negative streptococcus nephrons, which was an important critical value for clinical microbiology detection. The clinician should be informed immediately of this result. The result of culture after transfer was *N. gonorrhoeae*. Disseminated *N. gonorrhoeae* implies the spread of bacteremia and may produce immune complexes that lead to immune damage. Disseminated *N. gonorrhoeae* infections account for less than 1% of mucosal infections. Clinical manifestations include pyogenic arthritis and dermatitis.

Septic arthritis caused by *N. gonorrhoeae* is monoarticular or pauciarticular and is more commonly associated with positive synovial fluid cultures and negative blood cultures. Gonococcal bacteremia is more likely to be associated with polyarthralgia and skin lesions [15]. In this case, joint swelling and pain may be caused by hematogenous *N. gonorrhoeae* infection, but no clinical examination of synovial fluid was taken, so there is no evidence of etiology. Urine culture and vaginal secretion culture were examined. Even if urine sediment quantitation and urinalysis showed a WBC count of 356.4 (/µL), indicating urinary tract infection, *N. gonorrhoeae* was not cultured in urine and vaginal secretion of this patient, which may be related to antibiotics used clinically. The results of this case report are supported by existing studies of disseminated *N. gonorrhoeae* infection [16,17,18].

Bloodstream infection is a serious clinical infection [19]. The correct choice of antibiotics can improve the effective rate of bloodstream infection treatment. Some reports showed that tetracycline and ciprofloxacin are not suitable for the treatment of gonorrhea clinically [20,21], but cephalosporins and spectinomycin drugs can still be used for the treatment of gonorrhea [22,23]. Fever-Sanford Antimicrobial Treatment Guidelines recommended ceftriaxone + azithromycin as the first choice [24]. In this case, ceftriaxone was given for antimicrobial susceptibility treatment. Bacterial drug sensitivity test results in this case: ceftriaxone, cefotaxime, cefepime, and azithromycin were sensitive, ciprofloxacin was resistant, and tetracycline was mediated. Ceftriaxone 2 g for 24 h was selected according to clinical experience and treatment guidelines. After 7 days, blood culture was negative, joint swelling and pain were obviously improved, body temperature was normal, and the treatment was effective. She was discharged on December 31, 2023.

In conclusion, *N. gonorrhoeae* bloodstream infection is extremely rare in clinical cases. Normative and timely examination is helpful to improve the detection rate, and *N. gonorrhoeae* is mainly transmitted through sexual behavior, so urogenital specimens should be examined in time to identify the source of bacteria. Clinically, it is necessary to further strengthen the understanding of the pathogen and strengthen the communication and cooperation with the microbiology room, so as to realize the early diagnosis and effective treatment of the disease.
